# Battery‐Less Soft Millirobot That Can Move, Sense, and Communicate Remotely by Coupling the Magnetic and Piezoelectric Effects

**DOI:** 10.1002/advs.202000069

**Published:** 2020-05-16

**Authors:** Haojian Lu, Ying Hong, Yuanyuan Yang, Zhengbao Yang, Yajing Shen

**Affiliations:** ^1^ Department of Biomedical Engineering City University of Hong Kong Tat Chee Avenue Kowloon Hong Kong China; ^2^ Department of Mechanical Engineering City University of Hong Kong Tat Chee Avenue Kowloon Hong Kong China; ^3^ Shenzhen Research Institute of City University of Hong Kong Shenzhen 518057 China

**Keywords:** battery‐less sensing, soft millirobots, untethered motion, sensing

## Abstract

The soft millirobot is a promising candidate for emerging applications in various in‐vivo/vitro biomedical settings. Despite recent success in its design and actuation, the absence of sensing ability makes it still far from being a reality. Here, a radio frequency identification (RFID) based battery‐less soft millirobot that can move, sense, and communicate remotely by coupling the magnetic and piezoelectric effects is reported. This design integrates the robot actuation and power generation units within a thin multilayer film (<0.5 mm), i.e., a lower magnetic composite limb decorated with multiple feet imparts locomotion and a flexible piezoceramic composite film recovers energy simultaneously. Under a trigger of external magnetic guidance, the millirobot can achieve remote locomotion, environment monitoring, and wireless communication with no requirement of any on‐board battery or external wired power supply. Furthermore, this robot demonstrates the sensing capability in measuring environment temperature and contact interface by two different sensing models, i.e., carried‐on and build‐in sensing mode, respectively. This research represents a remarkable advance in the emerging area of untethered soft robotics, benefiting a broad spectrum of promising applications, such as in‐body monitoring, diagnosis, and drug delivery.

## Graphical abstract

The soft millirobot is a promising candidate for emerging applications in various in‐vivo/vitro biomedical settings. This work reports a radio frequency identification (RFID) based battery‐less soft millirobot that can move, sense, and communicate remotely by coupling the magnetic and piezoelectric effects, which benefits a broad spectrum of promising applications, such as in‐body monitoring, diagnosis, and drug delivery.

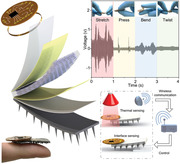

## 1 Introduction

Nature provides precious inspiration resources for scientists to design, manufacture and optimize robots. “Softness” is one salient feature exploited by most biological systems. Robots that adopt the soft nature attain better interaction to the environment, better adaptability to complex terrains and better self‐protection ability in extreme situations compared with traditional rigid robots.^[^ ^1^, ^2^, ^3^ ^]^ Indeed, benefiting from their versatility to sophisticated circumstances and a relatively large number of degrees of freedom, soft robots made of intrinsically soft material offer an exciting prospect of bridging the gap between rigid machines and soft humans.^[^ ^4^, ^5^ ^]^

Specifically, soft robots in small dimensions (from milli‐scale down to micro‐scale) are treated as promising candidates for emerging applications in various in‐vivo/vitro biomedical settings such as diagnosis, drug delivery, and targeted therapy.^[^ ^6^, ^7^, ^8^ ^]^ Recently, several small‐scale robots have been developed in a variety of shapes and structures for sophisticated functions, i.e., swimming in the liquid environment, walking on complex terrains, climbing over obstacles, crawling in limited space, loading heavy objects, etc..^[^ ^9^, ^10^, ^11^ ^]^ Such excellent capabilities of small‐scale soft robots build the essential foundation for tissue scaffolds construction,^[^ ^12^ ^]^ cell manipulation and characterization,^[^ ^13^ ^]^ drug delivery and target therapy,^[^ ^14^ ^]^ and low‐invasive surgery.^[^ ^15^ ^]^

Note that to make the soft robot be a reality, an effective sensing system is also essentially required rather than only actuation.^[^ ^16^, ^17^, ^18^ ^]^ Although sensing capability of macrosized soft robots has been designed and realized recently, the sensing capability of small soft robots is still very challenging because of the limited size for integration and signal transfer. Moreover, due to the tiny robot size and its harsh working environment, the requirements for a long lifespan power supply and wireless operations become further challenging.^[^ ^19^, ^20^, ^21^, ^22^, ^23^, ^24^ ^]^

To settle the preceding challenges of small‐scale soft robot/sensing integration, this paper reports a battery‐less soft millirobot that can move, sense, and communicate remotely by coupling the magnetic effect and the piezoelectric effect. Our smart robot, for the first time, integrates the power generation and actuation functions in a multilayer thin film (<0.5 mm), where a lower‐limb magnetic composite film decorated with multiple tapered feet is used to enhance motion and a unique piezoceramic composite film is used to recover energy simultaneously. As such, under the trigger of an external magnetic field, our robot can achieve combined locomotion, interface sensing, and wireless communication without any external electrical power input. The seamless integration of small‐scale soft robot and small‐scale sensing system represents a remarkable advance in the emerging area of untethered soft robotics.

## 2 Results

### 2.1 Design of the Untethered Soft Millirobot with a RFID Based Battery‐Less Sensing System

**Figure 1:**
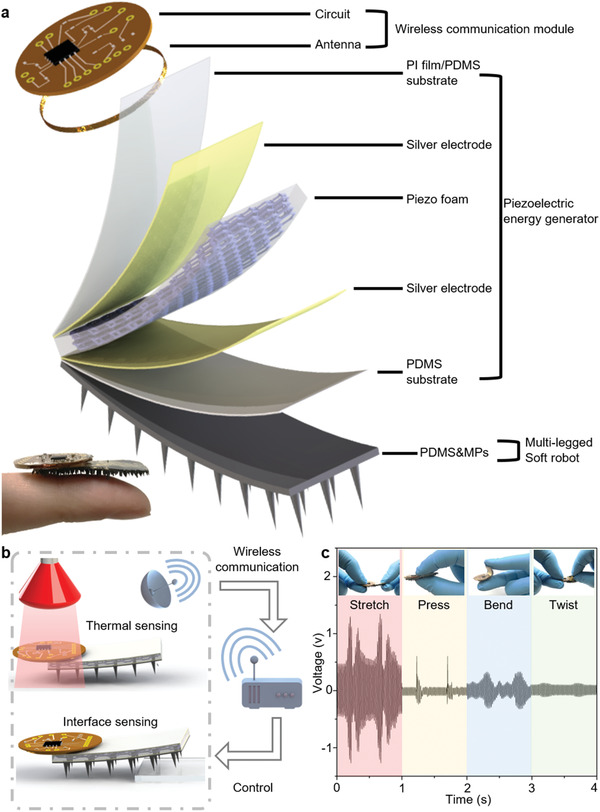
Multilegged, battery‐less, wireless sensing soft millirobot. a) Schematic diagram of the untethered milli‐scale soft robot with a RFID based battery‐less sensing system, showing three modules: wireless communication module, piezoelectric energy generator, and multilegged soft robot. b) Schematic illustration of the primary functions. The proposed multimodule robot system achieves a combined locomotion and tapping mode, where locomotion, interface sensing, and wireless communication can be executed concurrently. c) Voltage responses with the robot stretched, pressed, bended and twisted by human fingers. Considering the comparatively small actuation force provided by the magnetic field, we employ the bending‐deformation based locomotion type on this untethered milli‐scale soft robot.

Our untethered soft millirobot with a RFID based battery‐less sensing system is principally constructed of three modules, i.e., a top thin near‐field communication (NFC) electronic module, a middle piezoelectric energy generator (PEG) module, and a bottom multilegged soft robot (MSR) module (**Figure** **1**a). The prototype overall dimension is less than 10 × 30 mm^2^, comparable to a fingertip. Such robot in a millimeter scale is significant for biomedical application, which has attracted interests worldwide.^[^ ^3^, ^6^ ^]^ Advances in NFC technologies enable our wireless module (a flexible magnetic loop antenna and an IC) to acquire wireless signals and transmit data to any NFC‐enabled consumer devices, e.g., smartphones and computers. The middle part, i.e., the assembled multilayer PEG module, consists of a piezo foam composite (thickness ≈250 µm), two layers of electrodes (thickness ≈0.3 µm), and substrates (PI film with a thickness ≈100 µm and PDMS film with a thickness ≈30 µm), serving for the energy generation and interface sensing. The provided magnified scanning electron microscopic (SEM) images (Figure S1b, Supporting Information) of the piezo foam structures and surface patterns reveal a laminar ceramic framework with intense interconnections. The proverbial and well‐established connectivity of the piezoelectric phase dominates the unexceptionable piezoelectric property of the composite.^[^ ^25^ ^]^ The porous morphology of manufactured piezo foam framework, similar to the paper template, facilitates the infiltration of uncured Polydimethylsiloxane (PDMS) during the fabrication process, and endows the new piezo composite with outstanding flexibility, a property that has been constantly pursed but never achieved by piezoceramic materials. The lower‐limb magnetic robot module is decorated with multiple tapered legged structures. It is constructed of a ≈100 µm thickness PDMS and magnetic particle composite and ≈800 µm length (tip angle 40°) legs, reducing the surface contact area for soft robot locomotion (Figure S1c, Supporting Information).

The proposed multimodule design underpins our robot and enables a combined locomotion and tapping mode, where locomotion, interface sensing, and wireless communication can be executed concurrently (Figure 1a). Furthermore, the soft and flexible construction of the robot allows stretching, compressing, bending and twisting without any failure or fatigue (Figure 1c). Accordingly, the voltage signals are generated via the deformation of the PEG, where the output of the robot reaches its maximum value in the tensing state. Due to the thin multilayer design of the robot, the bending deformation is more likely to be realized during the robot locomotion, and a relatively smaller actuation force is required compared with that for tensing and compressing distortions. Therefore, the bending‐deformation locomotion type is applied to the untethered milli‐scale soft robot.

### 2.2 Manufacturing Process and Fundamental Characteristics of the Robot

**Figure 2:**
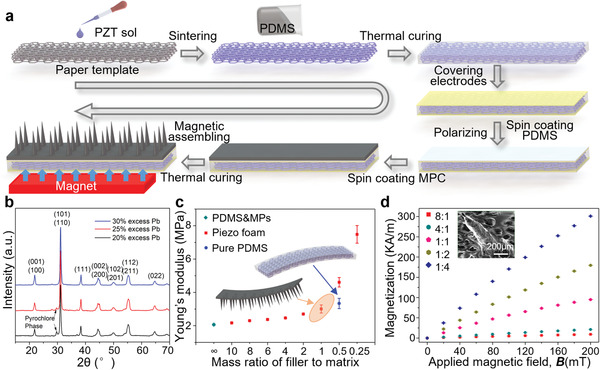
Manufacturing procedure and fundamental characteristics. a) Schematic illustration of the manufacturing procedures, including the preparation of the PZT foam precursor, the construction of the PZT foam framework, the fabrication of the PZT foam composite, and the appendix of the multilegged structure. b) X‐ray diffraction patterns of PZT foam framework with various excess Pb content during the preparation of PZT sol, showing that a 30% excess Pb will help to the formation of a pure perovskite structure of the PZT foam framework. c) Young's modulus of the multilegged soft robot and piezo foam composite with the various mass ratio of the PDMS matrix to the filler. Considering the strong combination of middle PEG module (the mass ratio of PDMS and PZT is 1:2) and bottom robot module, and also taking robot softness and actuation force into account, we select the mass ratio of PDMS and MPs of 1:1. d) The measured magnetization of the multilegged soft robot with different PDMS&MPs mass ratio, under an applied magnetic field, increasing from 0 to 200 mT.

Our robot is fabricated using a storey‐by‐storey overlay approach (**Figure** **2**a). The PEG module, based on the lead zirconate titanate (PZT) foam composite, is synthesized by the template‐assisted sol‐gel method.^[^ ^26^ ^]^ It mainly includes the following three steps: i) preparation of the precursor. The selected paper template is fully immersed by the prepared PZT sol and dried in an oven to get the precursor. ii) construction of a PZT foam framework. The PZT ceramic framework is obtained through sintering the prepared precursor (to dislodge the paper template). iii) fabrication of a PZT foam composite. The PZT foam framework is infiltrated with PDMS and cured in the oven. And then, silver electrodes are sputtered onto both sides of the PZT foam composite. The PEG fabrication is finalized with a PDMS film spin‐coated on its surface as the protective layer to realize the insulation property. After that, the lower‐limb MSR module is fabricated under the PEG layer via a modified magnetic (iron) micro particles (MPs) assisted molding approach.^[^ ^27^ ^]^ After spin coating a mixture containing magnetic micro particles & PDMS composite (MPC) on the middle PEG module, we apply an external magnetic field to form the multilegged structure during the solidification process. (Detailed fabrication process can be found in Figure S2 and Method S1 in the Supporting Information). Finally, a lab‐built flexible magnetic loop antenna and an NFC circuit are assembled on the top of the PEG layer. The prototype is 10 × 30 mm^2^, which is designed for alimentary canal at current stage, and it can be shrink down to a minuscule one by modifying the template. Both the PEG module and MSR module of the robot are scalable, which can be tailored to an arbitrary shape according to the practical requirements. And the NFC electronic module can be packed smaller via directly circuit writing on the top of the PEG layer, based on micro 3D circuit printing techniques.^[^ ^28^, ^29^, ^30^ ^]^

The X‐ray diffraction (XRD) patterns of the PZT foam framework with various excess Pb content indicate that a 30% excess Pb during the preparation of PZT sol helps the formation of a pure perovskite structure (Figure 2b), which is also confirmed through the Raman spectra (Figure S3a, Supporting Information). In addition, the energy dispersive spectroscopy (EDS) mapping (Figures S3b,c, Supporting Information) shows a homogeneous elements distribution of Pb, Zr, and Ti in the ceramic framework, without irrelevant elements. Apart from the characterization of PZT foam framework, the mechanical and magnetic property characterizations of bottom MSR module under various fabrication ratio (mass ratio of PDMS and MPs) are measured to make a compromise between the robot softness and actuation force (Figure 2c,d). The Young's modulus of the bottom multilegged soft robot varies from 2.17 to 7.48 MPa as the PDMS&MPs mass ratio increases from 10:1 to 1:4 (note that the measured reference Young's modulus of pure PDMS is ≈2.06 MPa), as depicted in Figure 2c (detailed results under ratio 1:1 can be found in Figure S4a in the Supporting Information). Under an applied magnetic field of 200 mT, the measured magnetization of the bottom MSR module increases from 7.09 to 300.85 kA m^−1^ as the PDMS&MPs mass ratio varies from 10:1 to 1:4, as manifested in Figure 2d (detailed results can be found in Figure S5 in the Supporting Information). The Young's modulus of the piezo foam composite (PZT and PDMS with mass ratio 2:1) is also characterized as ≈3.33 MPa, which is significantly lower than PZT ceramic (≈70 GPa). The relatively low Young's modulus and high fracture limit (Figure S4b, Supporting Information) allow the PZT foam composite to achieve different deformation (Figure 1c) without cracks. Considering the strong combination of middle PEG module and bottom robot module,^[^ ^31^ ^]^ and also taking robot softness and actuation force into account, we locate the optimal mass ratio range of PDMS and MPs between 1:1 and 1:2. Here 1:1 is used for prototyping.

**Figure 3:**
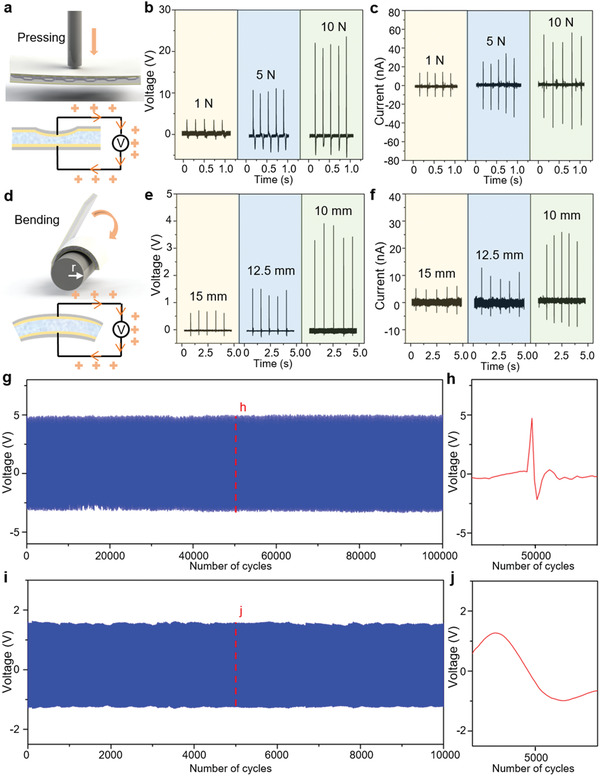
Piezoelectric responses of the piezo foam composite in different deformation modes. a) Schematic illustration of the tapping test achieved by a vibration generator. b,c) Output voltages (*V_impact_*) and currents (*I_impact_*) under different tapping forces with a frequency of 5 Hz. With the applied tapping force increasing from 1 to 10 N, the generated voltage and current increase accordingly. d) Schematic illustration of the bending test, with a PI film substrate of thickness 200 µm. e,f) Output voltages and currents under different bending curve radii, realized by a bending‐release process. The decrease in the curve radius leads to higher electric output (*V_bend_* and *I_bend_*). g) Output voltage in 100 000 tapping cycles under 1 N tapping force and 40 Hz tapping frequency, showing an excellent durability. h) Output voltages at the 50 000th tapping cycle, showing a typical sharp compressive response and relatively slow buildup. i) Output voltage of the piezo foam composite in 10 000 bending‐release cycles under 10 mm bending curve radius and 1 Hz bending frequency. j) Output voltage at the 5 000th bending cycle.

Regarding the locomotion mode of the soft magnetic robot, a series of piezoelectric tests have been designed to obtain the piezoelectric responses of the PZT foam composite under different deformation modes (theoretical analysis is in Figure S6 and Method S2 in the Supporting Information). In the tapping mode (**Figure** **3**a), when a compressive force is loaded under a tapping process with a frequency of 5 Hz, high open‐circuit voltage (*V*_impact_) and short‐circuit current (*I*_impact_) outputs occur (Figure 3b,c). With the applied tapping force rising from 1 to 10 N, the generated voltage and current increase accordingly (finger‐tapping measurement demonstration is shown in Video S1 in the Supporting Information). When the applied tapping force increases to 10 N, the output voltage and current rise up to 20 V and 50 nA, respectively, which are superior to PZT composites previously reported.^[^ ^32^ ^]^ With the tapping frequency increasing from 5 to 50 Hz, the generated voltage increases accordingly (Figure S6e, Supporting Information). The PZT foam composite also shows a high pressure sensitivity (10–30 mV kPa^−1^) in a large pressure range (kPa–MPa) with a good stability (Figure S6d, Supporting Information), corresponding to excellent ferroelectric hysteresis behavior shown by polarization‐electric field (P‐E) loops (Figure S6c, Supporting Information). Upon 100 000 tapping cycles, the output voltage of the PZT foam composite is kept unchanged, with a sharp compressive response and relatively slow buildup (Figure 3g,h). In the bending mode (Figure 3d), a PI film substrate with a thickness of ≈200 µm is selected, and the output open‐circuit voltage (*V_bend_*) and short‐circuit current (*I_bend_*) are measured under different curve radii (15, 12.5, and 10 mm). Once released, the generated output voltage and current of the robot prototpye are recorded (Figure 3e,f). The decrease of the curve radius leads to higher electric output (*V_bend_* and *I_bend_*), and the output voltage and current rise up to 3.5 V and 25 nA, respectively, when the curve radius decreases to 10 mm. Upon 10 000 bending‐release cycles, the output voltage of the PZT foam composite keeps nearly unchanged (Figure 3i,j).

### 2.3 Robot Locomotion Analysis and Wireless Sensing Demonstration

**Figure 4:**
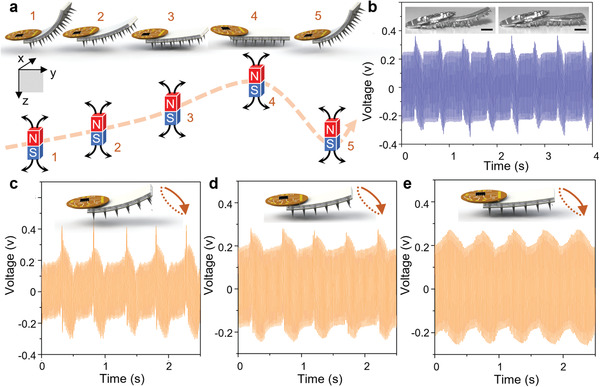
Robot locomotion and power generation in motion. a) Schematic illustration of the flap‐wave locomotion mode under the application of magnet with a reverse “V” trajectory in the *y‐z* plane. As the permanent magnet is moved upper and forward, the inflection angle of the robot decreases and the robot moves forward step by step until the robot's legs fully touch the ground. After the external magnetic field is off, the robot is back to the initial state under the pre‐deformation deflection. b) Corresponding output voltage during the robot locomotion in the flap‐wave mode, with a maximum output voltage up to approximately 0.36 V under the actuation frequency of 2 Hz. c–e) In‐situ tapping voltage response under the pre‐deformation angle of 30°, 20°, and 10°, respectively. The output voltage decreases with a small pre‐deformation angle. Scale bars, 5 mm.

When a PI film substrate (≈100 µm) with a pre‐deformation (≈20°) is applied, the robot exhibits a flap‐wave locomotion mode under the excitation of a magnet with a reverse “V” trajectory in the *y‐z* plane (**Figure** **4**a; Video S2, Supporting Information). Initially, under the circumstance where the magnet locates underneath the robot with a long distance, the robot holds the inflection posture. As the permanent magnet is moved upper and forward, the inflection angle of the robot decreases under the magnetic torque and gradient force, and the robot moves forward step by step until robot's legs fully touch the ground (basic theories about robot locomotion dynamic control can be found in Method S3 and Figure S7 in the Supporting Information). After the external magnetic field is turned off (magnetic bar moves downward and keep a long distance with the robot), the robot is back to the initial state under the pre‐deformation deflection. Meanwhile, electric output during the robot locomotion under flap‐wave mode is obtained in Figure 4b, where the maximum voltage output reaches up to approximately 0.36 V under the actuation frequency 2 Hz. In addition, under the excitation of the magnet with an “O” trajectory in the *y‐z* plane, the robot exhibits the in‐situ tapping pattern. The electrical output measurement under different pre‐deformation angles (30°, 20°, and 10°) have been measured (Figure 4c–e). Under the same circumstance of a magnetic field strength 100 mT and actuation frequency 2 Hz, the maximum voltage output reaches up to approximately 0.45 V with a 30° pre‐deformation angle, 0.28 V with a 20° pre‐deformation angle, and 0.27 V with a 10° pre‐deformation angle.

**Figure 5:**
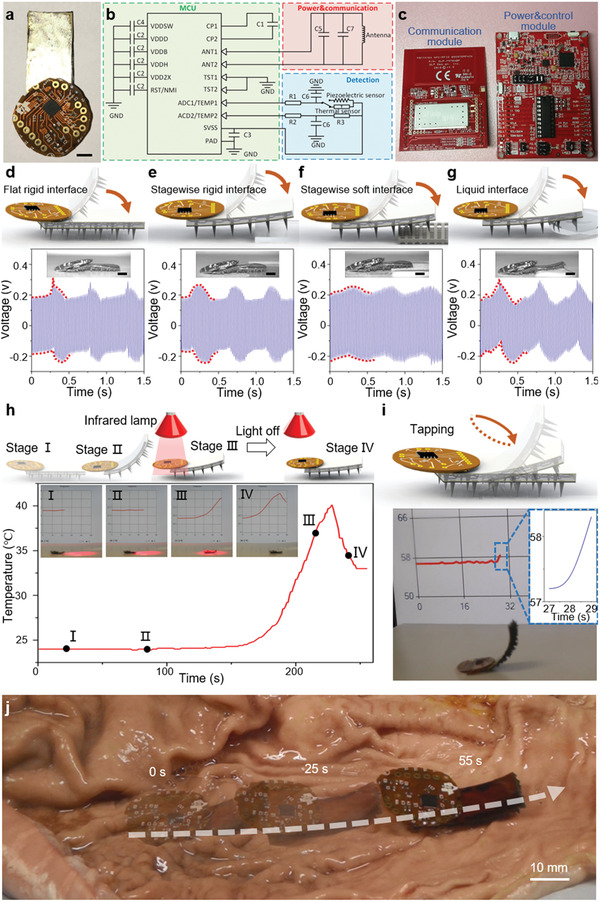
Demonstration of the robot locomotion and sensing capabilities. a) Optical image of the soft robot consists of a top thin electronic module, a middle PEG module, and a bottom actuation module. Scale bars, 5 mm. b) Detailed schematic diagram of the modified NFC circuit, including the microcontroller unit (MCU), the power & communication part, and the detection part. c) Optical image of the signal receiver module, including the communication module and the power and control module. d,e,f,g) Voltage responses in build‐in mode when the soft robot taps flat rigid interface, stagewise rigid interface, stagewise soft interface and liquid interface, respectively, showing different waveforms with various interfaces. Scale bars, 5 mm. h) Wireless thermal sensing with different signal feedback under four stages in carried‐on mode. When the robot approaches the infrared lamp lighting area from a distance, the sensor signal rises from ≈24 to ≈40 °C. i) Signal feedback of the in‐situ wireless tapping sensing on a flat rigid ground in carried‐on mode. There is an apparent signal increase when the robot taps the ground with a 2 Hz actuation frequency. j) Demonstration of robot locomotion capability on a cow stomach. The complex stomach internal structure with ropy gastric juice is selected, and the robot can move 65 mm in 55 s under actuation frequency 1 Hz at such a harsh in‐vivo simulated environment. Scale bar, 10 mm.

To demonstrate the sensing capability of the proposed soft robot, we characterized the connection interface and measured the environment temperature and by build‐in and carried‐on sensing modes. As shown in **Figure** **5**a, the soft robot with the wireless sensing ability consists of a top thin NFC electronic module (noted the signal receiver module is ≈10 cm underneath the robot), a middle PEG module, and a bottom MSR module. Figure 5b presents a detailed schematic illustration of the circuit depicted in Figure 5a, where the detailed description and the value of each electronic components can be found in Method S4 in the Supporting Information. The signal receiver module is shown in Figure 5c, where the left is for communication and the right part is for the board power supply and control.

To verify its interface‐sensing capability (Video S3, Supporting Information), we selected four different interfaces, i.e., flat rigid interface, stagewise rigid interface (glass sheet with thickness ≈1 mm), stagewise soft interface (foam sheet with thickness ≈2 mm) and liquid interface (with thickness ≈ 1 mm). Under the circumstance of the same magnetic field strength 100 mT, pre‐deformation angle 20°, and tapping frequency 2 Hz, the electric outputs of tapping flat rigid interface (Figure 5d), stagewise rigid interface (Figure 5e), stagewise soft interface (Figure 5f) and liquid interface (Figure 5g) can be represented with various waveform expression (detailed analysis can be found in Figure S8 and Method S5 in the Supporting Information). The voltage response resulting from bending motion (*V*_bend_) affects the waveform shapes during the whole bending period, while the voltage response resulting from the contact impact (*V*_impact_) is the main factor which causes the saltation peaks. When it comes to the flat rigid interface, the waveform exhibits a saltation peak at first due to the instantaneous impact and then decays gradually. When the magnetic field is off, the robot deforms back to the initial pre‐deformation state, and the waveform of output voltages exhibits a gradual peak from bending deformation (Figure 5d). Compared to the flat rigid interface, a gentler semi‐ellipse waveform without saltation peak is generated under a stagewise rigid interface due to the decrease of instantaneous impact between the interfaces (Figure 5e). Moreover, in the condition of a stagewise soft interface, the elastic contact between the soft robot and soft interface further decreases the impact force while increasing the impact time, leading to an extended gradual peak with lower voltage response (Figure 5f). In the last condition, influenced by the capillary force, a twin peak occurs when the robot taps a liquid surface (Figure 5g).

Apart from the build‐in sensing mode, the proposed robot is also able to perform carried‐on sensing mode, where the surrounding information is measured by the carried sensors on the robot. To demonstrate this capability, we measured the environment temperature during robot's walking (Figure 5h). In this operation, the robot is constructed with only the PDMS substrate without pre‐deformation, and a thermistor is attached on its body (Video S4, Supporting Information). Initially, the robot locates relatively far from the infrared lamp lighting area at stage I, and the temperature sensing feedback remains at ≈24 °C, noted that the sensor has been calibrated during the initialization. Under the trigger of an external magnetic (60 mT), a combined locomotion, i.e., the flap‐wave locomotion mode and the inverted‐pendulum locomotion mode^[^ ^27^ ^]^ are performed to move the robot forward into the infrared lamp lighting area (stage II). The thermal sensing feedback starts to increase up to ≈40 °C when the robot locates in the infrared lamp lighting area (stage III). In stage IV, the infrared lamp is turned off, and the thermal sensing feedback starts to decrease. Furthermore, we demonstrate that our robot can achieve wireless tapping sensing on the flat rigid ground (Figure 5i). Here the fabricated piezo composite, coupled with a PVDF substrate, serves as the strain sensing element. The deformation of the soft robot is measured, and its data is wirelessly exhibited by the GUI on a PC monitor (detailed description can be found in Method S4 in the Supporting Information). To further exhibit the potential applications in in‐vivo biomedical environment, we demonstrate the locomotion of our robot on a real cow stomach. The complex stomach internal structure with ropy gastric juice is selected, and the robot can move 65 mm in 55 s under actuation frequency 1 Hz in such a harsh in‐vivo simulated environment (Figure 5j; Video S5, Supporting Information). Through the measured signals feedback (Figure S9, Supporting Information), the twin peak waveforms caused by capillary force in the high viscosity wet surface can be detected easily. Moreover, the uneven surface of real cow stomach generates inhomogeneous waveforms during the testing. Considering the complex stomach internal structure with ropy gastric juice, the measured signal is tanglesome with noise existed and the surface variation cannot be explicitly distinguished via the waveform expression (both softness, wet and height coexist), which will be further studied in the future.

## 3 Discussion

Environment sensing ability is one of the most critical requirements for soft robots, but has scarcely been implemented at a small scale because of the small size and the power supply requirement. Here, we develop a RFID based battery‐less soft millirobot that can move, sense, and communicate remotely by coupling the magnetic effect and the piezoelectric effect. The integration of NFC wireless communication, flexible antenna, soft multiplayer piezoceramic‐magnet‐polymer composite, and the unique untethered magnetic actuation method underpins the proposed untethered milli‐scale soft robot with a fully autonomous operation.

The designed millirobot integrates the magnetic actuation and piezoelectric energy harvesting.^[^ ^33^ ^]^ As to the actuation, we employ the magnet‐driven method among all the remote actuation techniques owing to its inherent advantages in long‐range direct control and safe‐interaction with biological tissue.^[^ ^11^, ^27^, ^34^, ^35^ ^]^ The multiple‐leg design offers an effective body supporting and high motion agility of the robot. Although the piezo foam and wireless communication module influence the robot locomotion to some extent compared with previous design, the utilization of multiple‐leg structure still enhance the robot locomotion capacity in harsh environment.^[^ ^27^, ^36^ ^]^ In addition, benefiting from the designed multilegs, our robot exhibits a flap‐wave locomotion mode and an in‐situ tapping pattern by programming the magnetic field. As to the energy harvesting function, we choose to develop a thin flexible piezoelectric foam composite, considering that the piezoelectric effect has distinct merits in mechanical energy harvesting, sensing and actuation.^[^ ^31^, ^37^, ^38^, ^39^, ^40^, ^41^, ^42^ ^]^ To make the PEG module soft, thin and small, we propose a scalable paper‐template based PZT foam composite with the 3‐D interconnected ceramic framework. Such ultra‐thin piezoelectric ceramic‐polymer composite (≈250 µm) produces a high output voltage up to 20 V under simple finger tapping and demonstrate the similar softness as the PDMS basement.

In traditional ceramic‐polymer composites, the randomly dispersed low‐dimensional ceramic fillers severely influence the stiffness continuity and stress transfer, leading to severe degeneration of the piezoelectricity and flexibility.^[^ ^25^, ^26^, ^43^, ^44^, ^45^, ^46^ ^]^ In our method, the introduction of the 3‐D interconnected ceramic framework, originated from a paper fiber template, significantly improves the stress transfer and piezoelectric property, as a result of the increased connectivity of the piezo ceramic phase. Moreover, the stiffness continuity, attributed to the interconnected ceramic phase, dramatically reduces the equivalent Young's modulus of the piezoelectric foam composite (from 50–90 GPa to 3.33 MPa). The equivalent bending stiffness of the PEG module is thus hundreds of times smaller than that of commonly used piezoceramic materials, ensuring the agile locomotion of the robot even under the weak magnetic force excitations.

Through utilizing the new storey‐by‐storey overlay approach, the battery‐less sensing module and robot actuation module are integrated into a thin robot body (<0.5 mm). Benefiting from its whole‐PDMS composite, such a robot system is completely soft and flexible, which is resilient under stretching, pressing, bending and twisting loadings and can also generate corresponding piezoelectric responses with excellent durability. Under a remote fashion of the magnetic field control, our robot achieves the flap‐wave locomotion mode and in‐situ tapping sensing mode, where energy can be generated synchronously.

Our robot can realize the environment sensing by two different modes, i.e., the build‐in and carried‐on modes. For the build‐in mode, the environment is characterized by the interaction of the robot feet and the touching interface. Different interfaces will generate different contact forces and further lead to different electric signals. Such concept mimics the distinct mechanism widely used by the atomic force microscope (AFM) system. Though we here only demonstrate four different interface conditions to proof the concept, we believe that it can handle more complicate cases assisted by proper algorithms, such as disease diagnosis, morphology mapping, and physiological environment monitoring. For the carried‐on mode, the robot can work as a carrier to take most off‐the‐shelf sensors for various monitoring purposes. Benefiting from the multileg structure, our robot can carry ≈100 heavier load than itself.^[^ ^27^ ^]^ It offers a direct and effective solution to bridge the conventional sensing technique and millirobot. Moreover, the robot can effectively scavenge energy from motions and automatically send out signals out, offering limitless opportunities for the remote and long‐time health monitoring.

As demonstrated by the experiments, the untethered milli‐scale soft robot with the battery‐less sensing system offers a number of advantages, including entirely soft and flexible structure, long lifespan, excellent mechanical strength and reliability, energy autonomy, coinstantaneous locomotion & sensing & wireless communication and highly sensitive interface & thermal sensing. The concept of seamless integration of small‐scale soft robot sensing and energy harvesting provides a universal solution for the emerging untethered soft robotics on the millimeter scale, and it would be useful for a broad spectrum of applications such as untethered manipulation and sensing in poorly accessible space, and in‐vivo medical monitoring and therapy.

## Conflict of Interest

The authors declare no conflict of interest.

## Supporting information

Supporting Information

Video 1

Video 2

Video 3

Video 4

Video 5
