# Supplementary material for: Battery‐Less Soft Millirobot That Can Move, Sense, and Communicate Remotely by Coupling the Magnetic and Piezoelectric Effects
Source: Adv Sci (Weinh). 2020 May 16;7(13):2000069. doi: 10.1002/advs.202000069 (PMC7341101; doi:10.1002/advs.202000069)
Supplement: Supplementary file 1 — Supporting Information [file ADVS-7-2000069-s001.pdf]

Copyright WILEY-VCH Verlag GmbH & Co. KGaA, 69469 Weinheim, Germany, 2016.

Supporting Information

Battery-less soft millirobot that can move, sense, and communicate remotely by coupling the magnetic and piezoelectric effects

Haojian Lu, Ying Hong, Yuanyuan Yang, Zhengbao Yang, Yajing Shen**

This PDF file includes:

- Supplementary Figures
- Supplementary Methods
- Captions for Supplementary Videos S1 to S5
- References

Other Supplementary Materials for this manuscript includes the following:

- Supplementary Videos S1 to S5

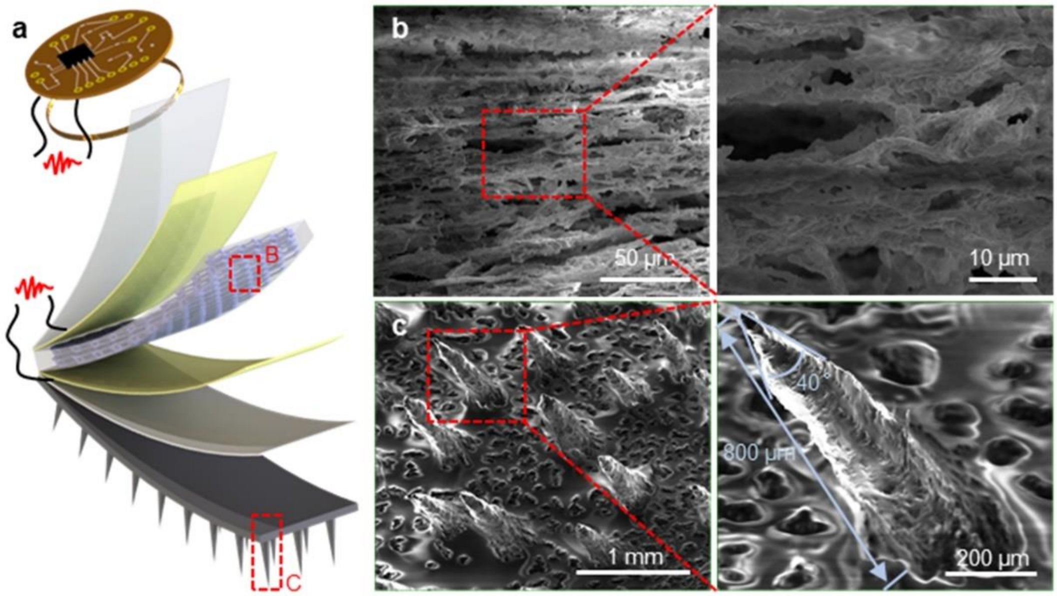

Figure S1. SEM images of the piezo foam framework and the robot leg structure. a) Schematic illustration of the location of SEM images. The PZT foam connects to the NFC chip electrically by two copper wires (bonded via silver conductive adhesive). b) SEM cross section images of the PZT ceramic foam, revealing an interconnected laminar ceramic framework with quantities of cavities. c) SEM images of the multi-legged structure, showing a series of milli-legs with ~ 800 μm length and ~ 40° tip angle.

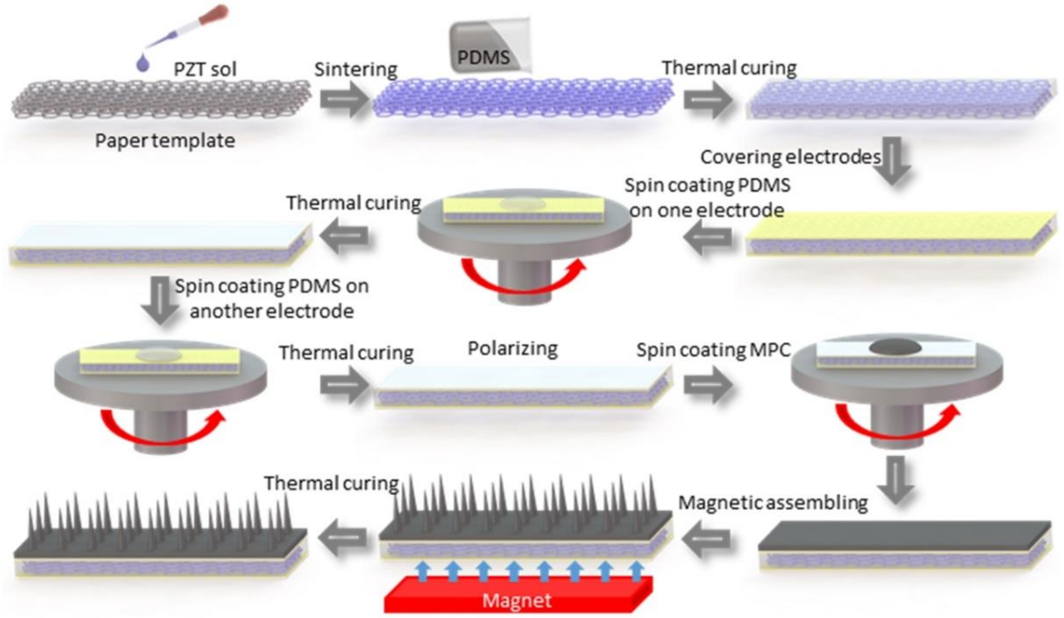

Figure S2. Detailed manufacturing procedures of the milli-scale multi-legged soft robot. The prepared PZT sol is dropped onto the paper until the whole paper is fully immersed by the sol. After dried, the fabricated precursor is sintered in high temperature to get the PZT ceramic framework. The fabricated PZT foam framework is filled with uncured PDMS and then cured in the oven. With silver electrode sputtered onto both sides of the PZT foam composite and PDMS film spin-coated as the protective layer, the fabricated PZT foam composite is polarized under an electric field. Then the bottom multi-legged soft robot module is fabricated based on a modified magnetic (iron) micro particles (MPs) assisted molding approach. After spin coating a mixture containing PDMS and MPs on the middle PEG module, an external magnetic field is applied during the solidification process to form the multi-legged structure.

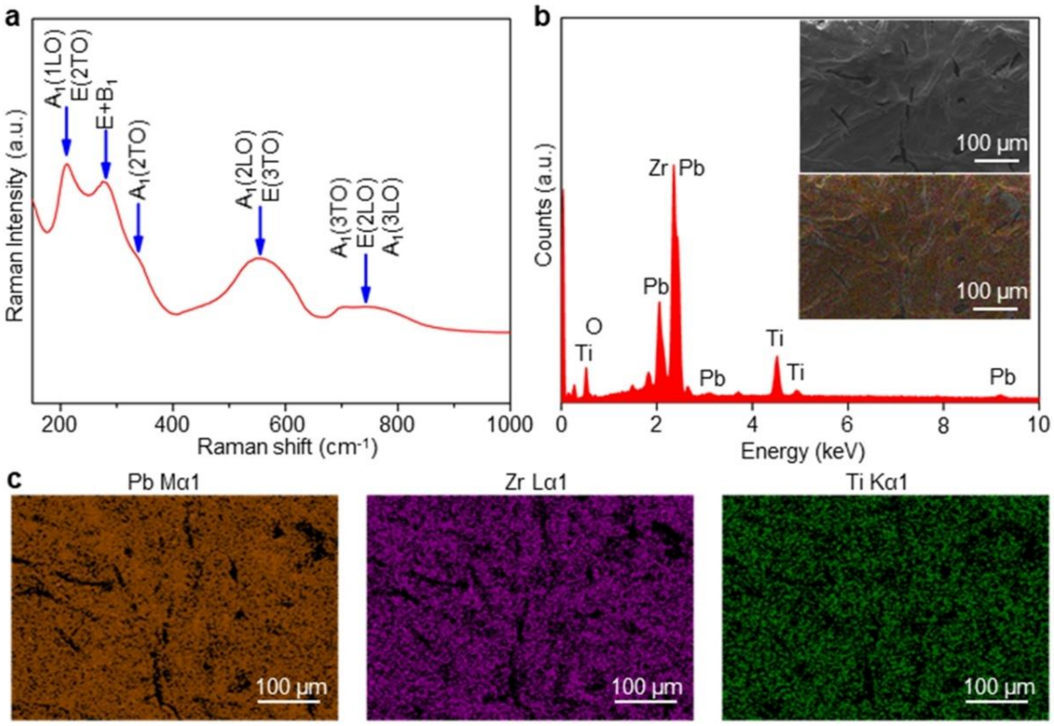

Figure S3. Fundamental characterization of the PZT ceramic framework. a) Raman spectra of the PZT ceramic framework with the introduction of 30% excess Pb, showing a perfect perovskite structure. b,c) Energy dispersive spectroscopy (EDS) mapping of the PZT ceramic framework, showing a homogeneous elements distribution of Pb, Zr, and Ti in the ceramic framework, without irrelevant elements.

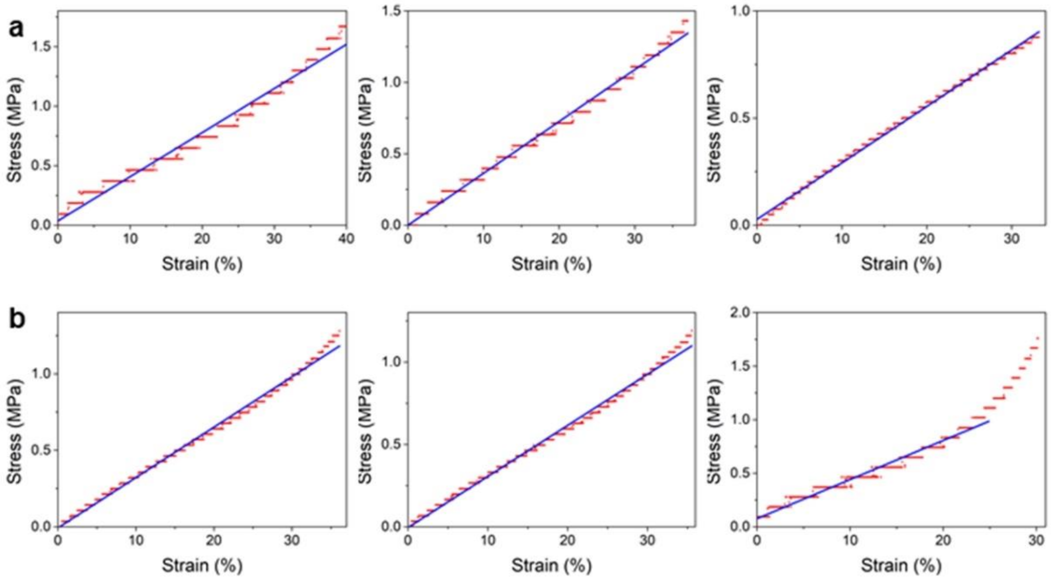

Figure S4. Young's modulus characterization. a) Young's modulus characterization of soft robot with fabrication ratio (mass ratio of PDMS and MPs) 1:1, three different specimens under same fabrication approach are selected. b) Young's modulus characterization of piezo foam composite, three different specimens under same fabrication approach are selected.

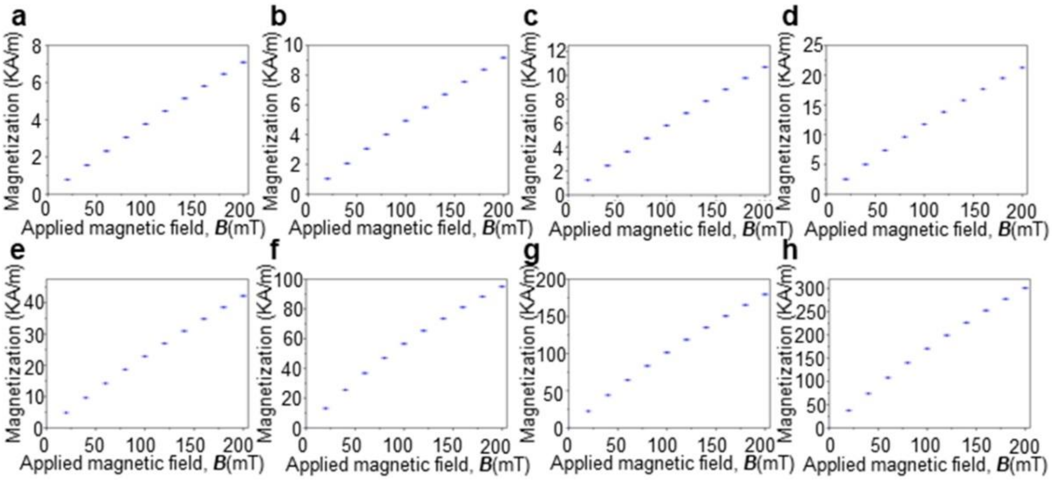

Figure S5. Measured magnetization of the multi-legged soft robot with various PDMS&MPs mass ratio. The applied magnetic field increases from 0 mT to 200 mT. a, b, c, d, e, f, g and h represent the PDMS&MPs ratio of 10:1, 8:1, 6:1, 4:1, 2:1, 1:1, 1:2 and 1:4 respectively.

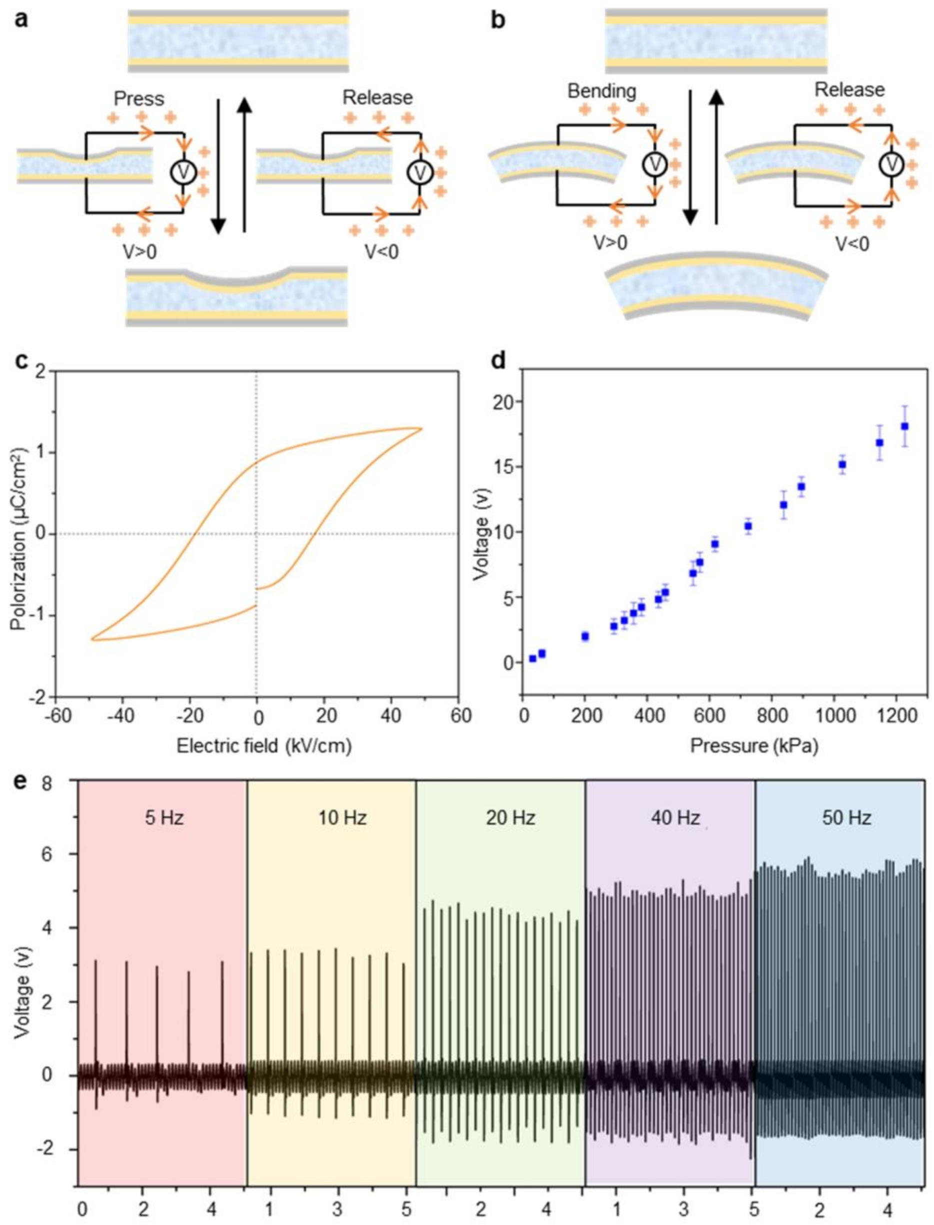

Figure S6. Piezoelectric mechanism and characterization. a) Schematic illustration of the theoretical shape for the tapping mode and the charge movement during press and release. b) Schematic illustration of the theoretical shape for the bending mode and the charge movement during bending and release. c) The polarization-electric field (P-E) loops of PZT foam composite measured with a frequency of 10 Hz, showing a good ferroelectric hysteresis behavior. d) Sensitivities of the PEG module under compression from 33 kPa to 1.2 MPa including two stages. In increasing stage (<600 kPa), the pressure sensitivity increases from 10.3 mV/kPa to 31 mV/kPa. In saturation stage (>600 kPa), the pressure sensitivity declines to 31 mV/kPa (~1.2 MPa). The load frequency is 5 Hz. e) Output voltages (V_{impact}) of the piezo foam composite under 1 N tapping forces with a different frequencies. With the tapping frequency increasing from 5 Hz to 50 Hz, the generated voltage increases accordingly.

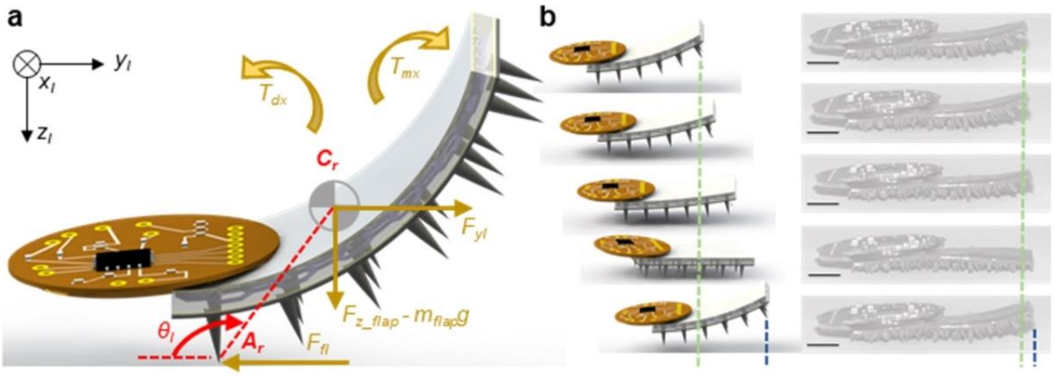

Figure S7. Robot locomotion analysis. a) Flap-wave locomotion modeling of the robot. To model the dynamics of the untethered multi-legged soft robot, we restrict modeling to a side-view of the robot in the *y*-*z* plane. The robot dynamic relations are developed with several parameters defined. b) The flap-wave locomotion mode of the soft robot with a pre-deformation deflection. Initially, the robot holds the inflection posture without magnetic field. As the permanent magnet is moved upper and forward, the inflection angle of the robot decreases under magnetic torque and gradient force, and the robot moves forward step by step until robot's legs fully touch the ground. After the external magnetic field is off, the robot is back to the initial state under the pre-deformation deflection. Scale bars, 5 mm.

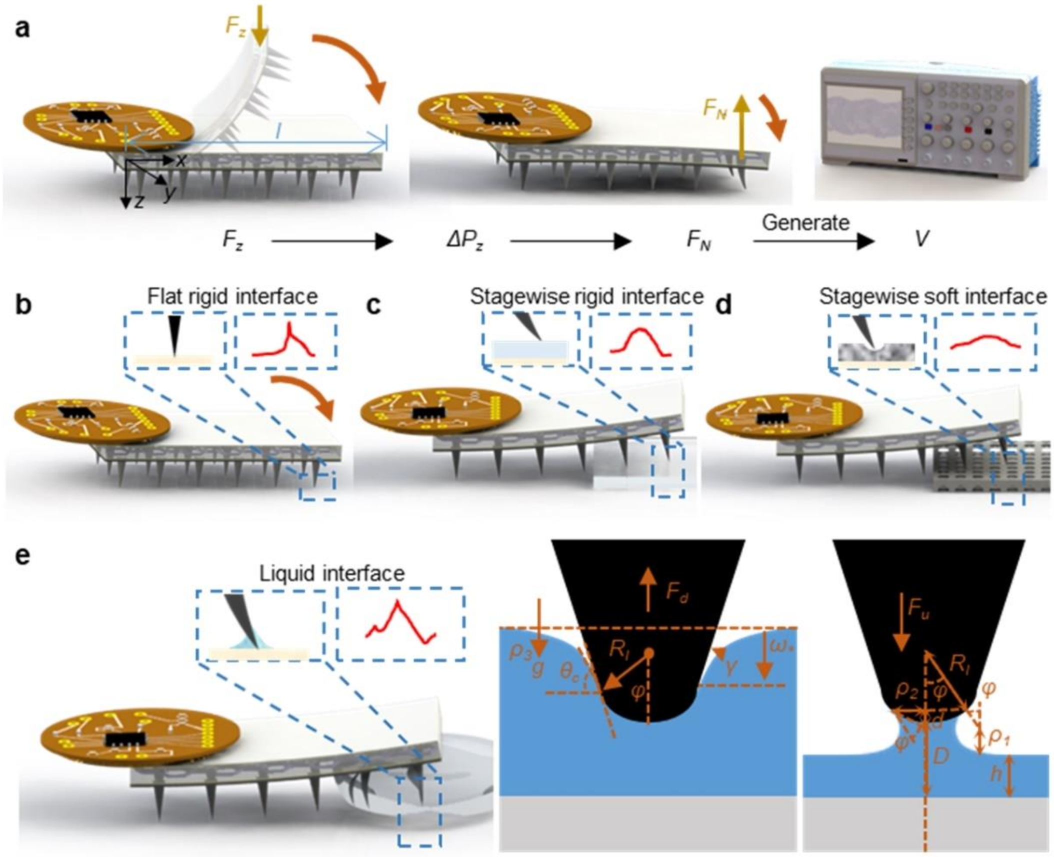

Figure S8. Interface-sensing analysis. a) Schematic illustration of the applied force on robot and the momentum conversion during the deformation process. b,c,d) The different waveforms of the output voltage when the soft robot gets contacted with flat rigid interface, stagewise rigid interface and stagewise soft interface, respectively. e) The capillary force when the robot contacts the liquid interface.

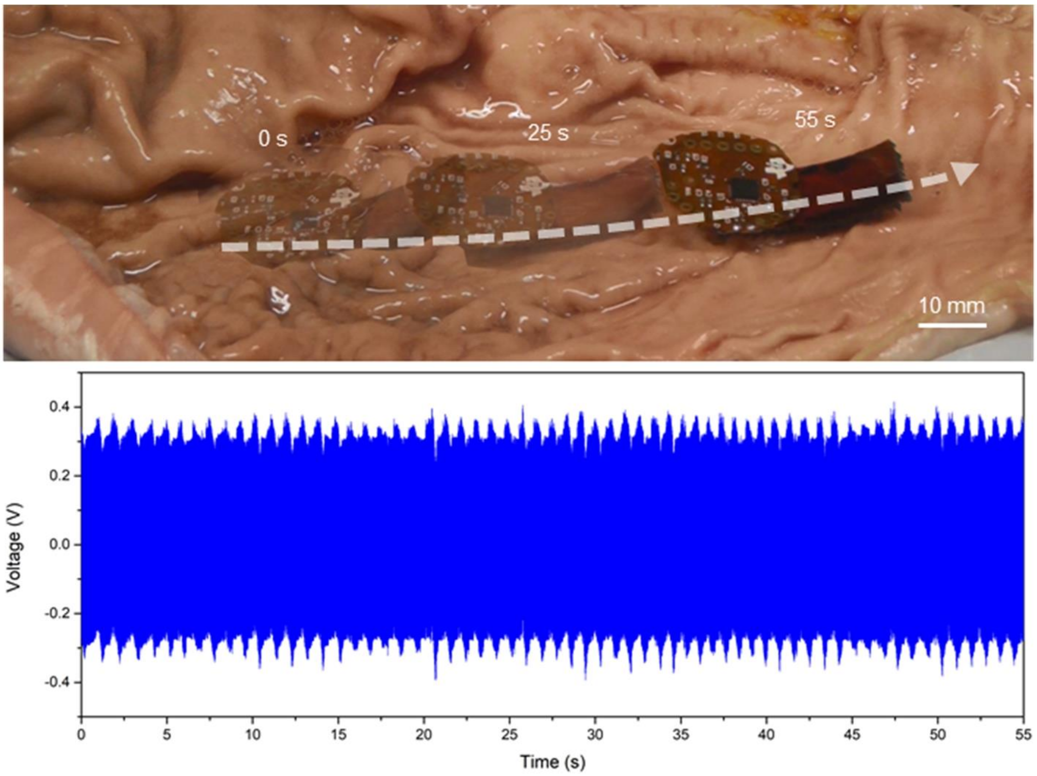

Figure S9. The measured signal s during the robot locomotion in a real cow stomach. The twin peak waveforms caused by capillary force in the high viscosity wet surface can be detected easily. Moreover, the uneven surface of real cow stomach generates inhomogeneous waveforms during the testing. Considering the complex stomach internal structure with ropy gastric juice, the measured signal is tanglesome with noise existed, and the surface variation cannot be explicitly distinguished via the waveform expression (both softness, wet and height coexist).

Supplementary Methods

1. Robot Manufacturing Process

The untethered milli-scale soft robot with a RFID based battery-less sensing system is fabricated via a combination of three modules, i.e., a top thin near-field communication (NFC) electronic module, a middle piezoelectric energy generator (PEG) module, and a bottom multi-legged soft robot (MSR) module (Figure 1a). The schematic illustration of the detailed fabrication process is shown in Figure S2 in the Supporting Information.

To fabricate the middle PEG module, the PZT [$Pb(Zr_{0.52}Ti_{0.48})O_3$] sol solution is firstly prepared, with lead (II) acetate trihydrate (Dieckmann, 99%), acetic acid (Dieckmann, 99%), zirconium (IV) propoxide (TCI, 70 wt% in 1-propanol) and titanium (IV) butoxide (TCI, 99%) as the starting material. The lead (II) acetate trihydrate with a 30% excess is mixed in acetic acid under vigorous stirring at 70℃ until the solid is fully dissolved. After cooling to room temperature, the zirconium (IV) propoxide and titanium (IV) butoxide are dropped slowly into the solution under stirring. Ultrasonic processing is then used to dissolve the possible aggregation during the mixing. The concentration of the final solution is 2 M, with a molar ratio of Pb:Zr:Ti=1.3:0.52:0.48. An excess of 30% Pb is used to compensate the lead loss due to its high volatilization in high temperature. And then, the prepared PZT sol is dropped onto a paper template with 0.5 mm thickness until the whole paper template is immersed by the sol. After dried at 60℃ for 1 hour, the sol immersed in paper converts to gel, showing an obvious stiffness increase. The precursor is then sintered in 1000℃ for 2 hours in the air atmosphere to remove the paper fiber template and finally get the PZT ceramic framework. Compared to the paper template, the sintered PZT ceramic framework keeps a similar morphology but shows a ~50% volume shrinkage. In the next step, to fabricate the PZT foam composite, the uncured PDMS (Sylgard 184, Dow Corning Co., Ltd) with 10wt% curing agent is prepared and then vacuumed to remove the bubble. The prepared uncured PDMS is poured onto the surface of

PZT foam framework until the whole PZT foam is fully infiltrated. After cured at 70℃ for 2h, a layer of silver film with a 300 nm thickness is sputtered onto both sides of the fabricated PZT foam composite as the electrodes. And then a layer of PDMS film is spin-coated onto the the silver electrodes as the protective layer. Finally, the PZT foam composite is polarized at 85℃ for 1 hour under the electric field of 15 kV/mm and then naturally cooled down to room temperature.

The bottom MSR module is fabricated on the middle PEG module based on a modified magnetic particle-assisted molding approach.[1-3] Firstly, the PDMS is prepared with base and curing agent ratio 10:1, and magnetic iron microparticles (MPs) with an average diameter of 6-10 μm (Alfa Aesar). After adjusting the pre-solution viscosity and weight ratio optimization, the weight ratio of 1:1 of the PDMS and MPs is selected (Figure 2c and 2d). Secondly, the solution is put on the middle PEG module and distributed well via spin coating. Thirdly, a permanent magnet with a size of 50 mm × 50 mm × 25 mm with surface magnetic intensity 0.25 T is put under the fabricated PEG module with distance 5 mm to form the multi-leg structures. Finally, the formed structure is then placed in a 7convection oven for thermal curing for 2 h at 70 °C to solidify.

After the fabrication of the PEG module and MSR module, a PI film substrate with thickness ~ 100 μm is attached to the side of PEG module to form the pre-deformation with various inflection angles. The PI film is attached to the PZT foam composite by uncured PDMS as the glue. Before curing, the PI film and PZT foam composite are attached to a 3D printed cylinder with different diameters. Once cured, the pre deformation of the robots with different curvatures is achieved, due to the inner stress between PI film and PZT foam composite. And then the reconstructed NFC module (a flexible magnetic loop antenna and sensor patch) is appended to the end surface using uncured PDMS.

2. Electrical Analysis of the PEG Module

The tapping test is conducted by a vibration generator, with a controlled oscillation frequency and taping force. The taping force is adjusted by the distance between the taping pillar and sample surface which can be detected and quantified by mechanical force sensors. For the bending test, the sample attached to PI film is first bent to fit the curved surface of a cylinder, with one end of the PI film tied onto the cylinder. Once released, the bent sample recovers to its initial form, producing an electric response. The cylinders we used is 3D printed with different diameters, which have various curve radii for the bending tests. For the cycling bending test in 10000 cycles, two ends of the PI film are fixed. When changing the distance of the two ends, the bending test with different curve radii can be achieved. The piezoelectric output voltage and current are measured by a digital oscilloscope (Rohde & Schwarz RTE1024) and a low-noise current preamplifier (Stanford Research SR570), respectively.

The electrical responses of the piezoelectric materials under mechanical deformation comes from the direct piezoelectric effect. In this study, we develop a new PZT foam composite, which shows a much better flexibility than the commonly-used PZT materials. For the taping mode as illustrated in Figure S6a in the Supporting Information, the piezoelectric response of the PZT foam composite follows the equation:[4]

$$V_{impact} = \frac{h_{PZT}}{A_{PZT}} g_{33} F_N \tag{S1}$$

where V_{impact} is the open-circuit output voltage under impact deformation, g_{33} is the piezoelectric voltage coefficient, F_N is the applied force, h_{PZT} and A_{PZT} are the thickness and area of the PZT foam composite, respectively. For the bending mode as illustrated in Figure S6B, the direct piezoelectric behavior is related to the stress (σ_{ij}), strain (ε_{ij}), electrical field (E_i), and electrical displacement (D_i).[4] For the plane-strain deformation ($\varepsilon_{22} = 0$) and

traction-free boundary condition ($\sigma_{33}=0$), the electrical displacement D_3 along the polarization direction x_3 is given by:

$$D_3 = \bar{e}\varepsilon_{11} + \bar{k}E_3 \tag{S2}$$

where $\bar{e} = e_{31} - (c_{13}/c_{33})e_{33}$ and $\bar{k} = k_{33} + (e_{33}^2/c_{33})$ are the effective piezoelectric constants, e_{ij} , c_{ij} , k_{ij} are the piezoelectric, elastic and dielectric parameters of PZT foam composite, respectively. The electrical displacement can be further written as:

$$D_3 = \bar{e}\varepsilon + \frac{\bar{k}V_{bend}}{h_{PZT}} \tag{S3}$$

where V_{bend} is the output voltage under bending deformation, ε is strain of PZT foam composite. The current I is related to the electric displacement, which can be written as:[5]

$$I = V_{bend}/R = -A_{PZT}\frac{dD_3}{dt} \tag{S4}$$

Combing Equation S3 and S4, we get

$$\frac{dV_{bend}}{dt} + \frac{h_{PZT}}{A_{PZT}R\bar{k}}V_{bend} = -\frac{\bar{e}h_{PZT}}{\bar{k}}\frac{d\varepsilon}{dt} \tag{S5}$$

The strain ε is the axial strain at the center of PZT foam composite, which is given by:[6]

$$\varepsilon = \left(\frac{\overline{EI}_{PI}}{\overline{EI}_{comp}}\right)\frac{H}{r} \tag{S6}$$

where H is the distance from the center of PZT foam composite to the neutral mechanical plane of the cross section, $1/r$ is the curvature of the PZT foam composite. $\overline{EI}_{PI}$ and $\overline{EI}_{comp}$ are the effective bending stiffness of PI film and multi-layer structure, respectively, which can be written as:[6]

$$\overline{EI}_{PI} = \left(\bar{E}_{PI}t_{PI}^3\right)/12 \tag{S7}$$

$$\overline{EI}_{comp} = \sum_{i=1}^{n}\bar{E}_i t_i\left[t_i^2/3 + \left(\sum_{j=1}^{i}t_j - y_{neutral}\right)\left(\sum_{j=1}^{i}t_j - y_{neutral} - t_i\right)\right] \tag{S8}$$

where $i=1$ represents the *1st* layer in the structure, $\bar{E}_i$ and t_i are the plane-strain modulus and thickness of the *ith* layer, and

$$y_{neutral} = \left[\sum_{i=1}^{n} \bar{E}_i t_i \left(2\sum_{j=1}^{i} t_j - t_i \right) \right] \Big/ \left(2\sum_{i=1}^{n} \bar{E}_i t_i \right) \quad \text{(S9)}$$

is the location the neutral mechanical plane.

3. Robot Locomotion Analysis

When the permanent magnet is applied with a reverse "V" trajectory in *y*-*z* plane, as depicted in Figure 4a, the untethered mill-scale soft robot exhibits the flap-wave locomotion mode (Videos S2, Supporting Information). At the initial state, when the magnet is located underneath the robot with a long distance, the robot with the pre-deformation angle holds the inflection posture. When the permanent magnet is controlled to move obliquely upward, the inflection angle of the robot decreases under magnetic torque and gradient force. The front legs of the robot are fall down to align the magnetic induction line, and the robot is dragged forward to local maximum magnetic field. The robot moves forward step by step until robot's legs are fully touching the ground. After the permanent magnet is controlled to move downward, the robot is back to the initial state under the pre-deformation deflection. To analyze the flap-wave locomotion mode, the robot is mapped in a 2D plane (y_l-z_l). Therefore, the robot dynamic model can be represented as:

$$m_r \ddot{C}_{ry} = F_{my} - F_{fl} \quad \text{(S10)}$$

$$m_r \ddot{C}_{rz} = F_{mz} + m_r g \quad \text{(S11)}$$

$$J_r \ddot{\theta}_l = T_{mx} + F_{fl} \cdot \left| \mathbf{C_r} - \mathbf{A_r} \right| \cdot \sin\theta_l - T_{dx} \quad \text{(S12)}$$

$$C_{ry} = A_{ry} - \left| \mathbf{C_r} - \mathbf{A_r} \right| \cos\theta_l \quad \text{(S13)}$$

$$\ddot{C}_{ry} = \ddot{A}_{ry} + \left| \mathbf{C_r} - \mathbf{A_r} \right| \ddot{\theta}_l \sin\theta_l - \left| \mathbf{C_r} - \mathbf{A_r} \right| \dot{\theta}_l^2 \cos\theta_l \quad \text{(S14)}$$

$$C_{rz} = A_{rz} + |\mathbf{C_r} - \mathbf{A_r}| \sin\theta_l \tag{S15}$$

$$\ddot{C}_{rz} = \ddot{A}_{rz} + |\mathbf{C_r} - \mathbf{A_r}| \cos\theta_l - |\mathbf{C_r} - \mathbf{A_r}| \dot{\theta}_l^2 \sin\theta_l \tag{S16}$$

where $\mathbf{C_r}[C_{rx} \quad C_{ry} \quad C_{rz}]$ represents the robot's center of mass (CoM) coordinate, which can be calculated via:

$$\mathbf{C_r}[C_{rx} \quad C_{ry} \quad C_{rz}] = \left[\frac{\sum_{i=1}^{n} m_{ri} C_{rxi}}{\sum_{i=1}^{n} m_{ri}} \quad \frac{\sum_{i=1}^{n} m_{ri} C_{ryi}}{\sum_{i=1}^{n} m_{ri}} \quad \frac{\sum_{i=1}^{n} m_{ri} C_{rzi}}{\sum_{i=1}^{n} m_{ri}} \right] \tag{S17}$$

and $\mathbf{A_r}[A_{rx} \quad A_{ry} \quad A_{rz}]$ represents the coordinate of the point that the robot attaches the ground. J_r is the polar moment of inertia of the robot, calculated as $J_r = \sum \Delta m_{ri} D_{ri}^2$, where D_{ri} is the distance between the m_{ri} and the spindle. m_r is the mass of the robot and g is the gravity acceleration. $\ddot{C}_{ry}$ and $\ddot{C}_{rz}$ represent the robot acceleration along *y* axis and *z* axis respectively. F_{my} and F_{mz} represent the magnetic force the robot experienced along *y* axis and *z* axis respectively. F_{fl} is the robot sliding friction force, θ_l and $\ddot{\theta}_l$ represent the robot orientation angle and angle angular acceleration respectively, T_{mx} and T_{dx} are the magnetic torque and deflection torque the robot experienced.

4. Wireless Electronic Module in Carried-on Mode

The wireless perception function of the soft robot in carried-on mode is achieved by a single chip NFC/RFID field powered system with the RF430FRL152H sensor patch from Texas Instruments Inc.

The whole system mainly consists of three parts: the microcontroller unit (MCU), the power & communication part, and the detection part. The microcontroller unit is the core part that connects the antenna and the detection part. The antenna can supply power to the MCU from the DLP7970ABP by using the protocol ISO15693 in a high frequency of 13.56 MHz.

After the MCU get powered, it will activate the VDD (operating voltage of inside device), RST (resetting MCU if problems arise), as well as the detection part. The detection part is responsible for sensing the change in the external environment and then converting it to an electric signal.

The whole circuit is battery-less, which means that there is no power supply in the circuit. And the antenna is assigned with two functions, harvesting RF power and transmitting signals. There are mainly three steps for the whole working process of the circuit. First, the antenna receives the power and then stabilizes the current to power the MCU through the impedance matching method. And the capacitor (C_5=3.9 pF and C_7=33 pF) connected to the antenna is used to hold the voltage to power the MCU. Second, the powered MCU activates VDD and RST, receiving the signals 4 times per second (the sensing time can be changed via the over-the-air programming method). Finally, once signals generated, the antenna transmits the data back to the reader. All the values of other resistors and capacitors are listed as following: R_1=1 kΩ, R_2=1 kΩ, R_3=100 kΩ, C_1=0.01 μF, C_2=0.1 μF, C_3=1 μF, C_4=2.2 μF, C_5=3.9 pF, C_6=22 pF, and C_7=33 pF.

In this study, to monitor the motion mode and external temperature change, we choose a piezoelectric sensor and a temperature sensor. The piezoelectric sensor is the PZT foam composite we fabricated, which can produce different electric signals under different motion mode of the soft robot. The thermistor is the main part of the temperature sensor, which reacts to the external temperature change. A calibrate resistor (R_3=100 kΩ) is used to calibrate the electric signals produced by the sensors.

For the external temperature change detection, when the electric signal produced from thermistor is transferred to the signal receiver through a wireless way, there is an apparent signal change on the PC monitor, which can reflect the temperature change around.

For the robot locomotion detection, the coupled PVDF substrate can not only move the stress-neutral plane, but also provide additional power supply besides the PZT foam composite, both of which increase the voltage signal from the bending locomotion. When the piezoelectric signal produced from robot deformation is transferred to the signal receiver through a wireless way, there is an apparent signal increase on the PC monitor when the robot taps the ground with a 2 Hz actuation frequency. However, due to the low sampling frequency (4 times per second), the piezoelectric signals produced from robot tapping is hard to be collected in each tapping cycle, which needs to be improved in our future work.

5. Interface-sensing Analysis in Build-in Mode

Under the same magnetic field strength of 100 mT, same pre-deformation angle of 20°, and same tapping frequency of 2 Hz, the voltage outputs of tapping flat rigid interface (Figure 5d), stagewise rigid interface (Figure 5e), stagewise soft interface (Figure 5f) and liquid interface (Figure 5g) can be represented with various waveform expression. Here we only consider the major influence of bending strain and impact force on the piezoelectric response, solving the simplified formulae to get the relationship between the piezoelectric response and robot motion.

To further explain the waveform shapes in Figure 4c-4e and Figure 5d-5g, we need to introduce the influence of the impact when the robot touches the ground, as well as the bending strain through the whole movement process. The piezoelectric output V_{out} is represented as:

$$V_{out} = V_{impact} + V_{bend} \tag{S18}$$

where V_{impact} and V_{bend} are the output voltages from impact force and bending strain respectively.

a) The output voltages from bending strain V_{bend}

For the initial condition $V_{(t\,=\,0)} = 0$, The solution of Equation S5 can be given by[4]

$$V_{bend} = \frac{\bar{e} h_{PZT} H \overline{EI}_{PI}}{\bar{k}\,\overline{EI}_{comp}} e^{-\frac{h_{PZT}}{A_{PZT}\bar{k}} t} \int_0^t \frac{r^2 dr}{dt} e^{\frac{h_{PZT}}{A_{PZT} R \bar{k}} t} dt \tag{S19}$$

When we consider the whole composite structure as a simple cantilever beam under a single force F_z, according to the Euler–Bernoulli beam theory, the deformation at x in the z axis can be written as:

$$z = -\frac{F_z x^2}{6\overline{EI}_{comp}}(3l - x) \tag{S20}$$

where x is the distance from the base along x axis and l is the length of the whole PZT foam composite. As a result, the curvature radius $r = \frac{\left[1 + (\frac{dz}{dx})^2\right]^{3/2}}{\left|\frac{d^2 z}{dx^2}\right|}$ can be given as

$$r = -\frac{F_z\left[1 + 9(2lx - x^2)^2\right]^{3/2}}{36\overline{EI}_{comp}(l - x)} \tag{S21}$$

b) The output voltages from impact force V_{impact}

During the bending process under the magnetic field, the motion impact applied to the robot along z axis can be expressed as the integral of force over time (Figure S8a in the Supporting Information), which is

$$I_{mov} = \int_0^{t_\theta} F_z dt \tag{S22}$$

where I_{mov} is the motion impact applied to the soft robot along z axis, t_θ is the time when the robot moves to the orientation angle θ, and F_z is the applied force along z axis, calculated as $F_z = F_{mz} + m_r g$. According to the theorem of momentum,

$$\Delta p_z = I_{mov} = \int_0^{t_\theta} F_z dt \tag{S23}$$

where Δp_z is the momentum change of the robot along *z* axis from the initial state to t_θ. When the soft robot gets contacted with the interface, the momentum decreases, converting to the instantaneous impact I_{int} between the interfaces, which can be represented as:

$$-\Delta p_z = I_{\text{int}} = F_N \Delta t \tag{S24}$$

where F_N is the impact force between the interfaces, and Δt is the impact time. To simplify the calculation, we assume that F_N keeps constant at the impulse process and equals to the load force applied to the piezo foam composite. Once contacted, the piezoelectric output V_{impact} is generated. According to Equation S1, V_{impact} is represented as:

$$V_{impact} = -\frac{h_{PZT}}{A_{PZT}} g_{33} \frac{\int_0^{t_\theta} F_z dt}{\Delta t} \tag{S25}$$

In the deformation period when the robot deforms from a certain pre-deformation angle, the inflection angle of the robot decreases while the radius of curvature increases until the robot becomes fully flat. According to Equation S19, larger pre-deformation angle will result in larger bending voltage intensity, which corresponds with the experimental results in Figure 4c.

The voltage response resulting from bending motion (V_{bend}) affects the waveform shapes during the whole bending period, while the voltage response resulting from the contact impact (V_{impact}) is the main factor which causes the saltation peaks. As a result, the introduction of stagewise rigid interface decreases V_{bend} and V_{impact} compared to the flat rigid interface, while the introduction of stagewise soft interface further causes the decrease of V_{impact} compared to the stagewise rigid interface. Furthermore, a liquid interface will introduce the capillary force, leading to a twin peaks (V_{impact}) when tapping on a liquid surface. Extended explanation is shown as following:

I) Flat rigid interface (Figure S8b, Supporting Information). When the magnetic field is applied, the inflection angle of the robot decreases until the robot fully touches the ground.

The waveform exhibits a saltation peak at first, and then decays gradually. When the magnetic field is off, the robot deforms back to the initial state under the pre-deformation deflection and the waveform exhibits a gradual peak. The saltation peak is the result of the instantaneous impact I_{int} when the soft robot gets contacted with the rigid interface. In contrast, when the robot deforms back to the initial pre-deformation state, the saltation peak disappears.

II) Stagewise rigid interface (Figure S8c, Supporting Information). Compared to the flat rigid interface, there is no saltation peak in the waveform for the stagewise rigid interface due to the decrease of instantaneous impact between the interfaces. The existence of the stage leads to the decrease of the orientation angle, which means that the motion time t_θ of the soft robot also decreases at the same time. As a result of that, the voltage response from impact force V_{impact} will be decreased according to the Equation S25. And according to Equation S18, the output voltage V_{out} is mainly determined by the change of V_{impact} due to nearly constant V_{bend}. As a result, the introduction of stagewise rigid interface decreases the output voltage compared to the flat rigid interface.

III) Stagewise soft interface (Figure S8d, Supporting Information). In this condition, the tapping results exhibit a further more gently gradual peak compared with the two conditions mentioned. The existence of stagewise soft interface increases the impact time Δt, thus the output voltage V_{out} will be decreased according to Equation S18 and S25. Moreover, due to the increase of impact time, we get an extended gradual peak in the waveform.

IV) Liquid interface (Figure S8e, Supporting Information). Different from the three conditions above, a liquid interface will introduce the capillary force. A twin peak occurs when tapping on a liquid surface. When the robot pats down and contacts the liquid interface under the magnetic force and torque, the capillary force exhibits. We take the single leg as an example, and the single leg tip capillary force can be represented as:[7-9]

$$F_d = \rho_3 g R_l^2 \left(2 \frac{\omega_*}{R_l} \sin\varphi - \varphi + \sin\varphi\cos\varphi \right) - 2\gamma \sin\theta_c \quad \text{(S26)}$$

where we consider the cross section of robot's leg tip as a circle with radius R_l. And ρ_3, g, ω_*, φ, γ and θ_c represent the liquid density, gravitational acceleration, the height of the contact line above the undisturbed free surface, contact angle, interfacial tension and angle of the interface with the horizontal at the contact line respectively.

When the robot is lifted up and break away from the liquid interface under the pre-deformation angle deflection, the capillary force also exhibits. The pressure in the liquid is less than the atmospheric pressure based on the Young–Laplace equation, which can be represented as:[10]

$$\Delta p = \sigma \left(\frac{1}{\rho_1} - \frac{1}{\rho_2} \right) \approx \frac{\sigma}{\rho_1},\ \rho_1 \ll \rho_2 \quad \text{(S27)}$$

where σ, ρ_1 and ρ_2 represent the surface tension, a lesser curvature radius of the liquid surface and robot leg tip –liquid contact area radius. For the reason that such pressure applied to the leg tip-liquid interface has $\pi\rho_2^2 \approx 2\pi R_l d$, the single leg tip capillary force can be represented as:

$$F_u = 2\pi R_l d \frac{\sigma}{\rho_1} = \frac{2\pi R_l \sigma (1 + \cos\varphi)}{1 + \frac{D - h}{d}} \quad \text{(S28)}$$

where we assume that the contact angles φ is same for robot leg tip and the liquid, which leads to $\rho_1 \cos\theta + \rho_1 = D + d - h$. D, d and h represent the robot leg tip-substrate separation, immersion depth, and the liquid film thickness.

References

[1] J. V. I. Timonen, C. Johans, K. Kontturi, A. Walther, O. Ikkala, R. H. A. Ras, *Acs Appl Mater Inter* **2010**, 2, 2226.

[2] M. Cao, J. Jie, L. Kan, S. Dou, J. Lei, *Adv Funct Mater* **2014**, 24, 3235.

[3] H. Lu, M. Zhang, Y. Yang, Q. Huang, T. Fukuda, Z. Wang, Y. Shen, *Nat Commun* **2018**, 9, 3944.

[4] Y. Su, L. Shuang, H. Yong, L. Rui, Z. Zhang, P. Joe, C. Dagdeviren, *Extreme Mech Lett* **2017**, 15, 10.

[5] D. Canan, Y. Byung Duk, S. Yewang, P. L. Tran, J. Pauline, A. Eric, X. Jing, D. Vijay, D. Behrooz, F. Xue, *P Natl Acad Sci USA* **2014**, 111, 1927.

[6] C. R. Bowen, H. A. Kim, P. M. Weaver, S. Dunn, *Energ Environ Sci* **2014**, 7, 25.

[7] J.-S. Koh, E. Yang, G.-P. Jung, S.-P. Jung, J. H. Son, S.-I. Lee, P. G. Jablonski, R. J. Wood, H.-Y. Kim, K.-J. Cho, *Science* **2015**, 349, 517.

[8] D. Vella, *Langmuir* **2008**, 24, 8701.

[9] J. B. Keller, *Phys Fluids* **1998**, 10, 3009.

[10] J. N. Israelachvili, *Intermolecular and surface forces*, Academic press, **2011**.

Caption for Supplementary Videos

Video S1

Voltage responses of the piezo foam composite under the excitation of finger tapping. The video shows the output voltages of the piezo foam composite under finger tapping with a frequency of 7 Hz. When the tapping force increases from the finger, the output voltages also increases at the same time and finally reach up to ~ 20 V (5 V per lattice). The video is at 1× playback speed.

Video S2

The flap-wave locomotion of the soft robot. The video shows the flap-wave locomotion of the multi-legged soft robot with a pre-deformation deflection. This motion mode is generated by a reverse "V" trajectory of magnet with surface magnetic field intensity 100 mT on *y*-*z* plane. This untethered millirobot with flexible tapered feet moves 5 mm in 4 s with frequency 2 Hz. Meanwhile, electric output during the robot locomotion under flap-wave mode can be obtained, where the maximum output voltage can reach up to approximately 0.36 V under the actuation frequency of 2 Hz. The video is at 0.25× playback speed.

Video S3

Interface-tapping sensing in build-in mode. The video shows the output voltages when the soft robot taps flat rigid interface (first), stagewise rigid interface (second), stagewise soft interface (third) and liquid interface (fourth), respectively. This motion mode is generated by a "O" trajectory of magnet with surface magnetic field intensity 100 mT on *y*-*z* plane. The waveforms of the output voltages are various for different interfaces. Corresponding analysis can be found in Text S6. The video is at 0.25× playback speed.

Video S4

Thermal sensing in carried-on mode. The second video shows the wireless thermal sensing in carried-on mode with different signal feedbacks when the robot approaches the infrared lamp lighting area from a distance. The millirobot moves 60 mm in 120 s with frequency 2 Hz, and the thermal signal changes from 24℃ to 40℃. After the infrared lamp is off, the thermal signal decreases along with the temperature decrease. The video is at 10× playback speed.

Video S5

Cow's stomach locomotion demonstration. The video shows locomotion adaptability of the robot. The complex stomach internal structure with ropy gastric juice is selected, and the robot can move 65 mm in 55 s under actuation frequency 1 Hz at such a harsh in vivo simulated environment. The video is at 10× playback speed.
